# A lightweight noise-tolerant encryption scheme for secure communication: An unmanned aerial vehicle application

**DOI:** 10.1371/journal.pone.0273661

**Published:** 2022-09-19

**Authors:** Arslan Shafique, Abid Mehmood, Mourad Elhadef, Kashif Hesham khan

**Affiliations:** 1 Department of Electrical Engineering, Riphah International University, Islamabad, Pakistan; 2 Department of Computer Sciences, Abu Dhabi University, Abu Dhabi, UAE; 3 Department of Computer Sciences, RMIT University, Melbourne, Australia; Beijing Institute of Technology, CHINA

## Abstract

In the modern era, researchers have focused a great deal of effort on multimedia security and fast processing to address computational processing time difficulties. Due to limited battery capacity and storage, Unmanned Aerial Vehicles (UAVs) must use energy-efficient processing. In order to overcome the vulnerability of time inefficiency and provide an appropriate degree of security for digital images, this paper proposes a new encryption system based on the bit-plane extraction method, chaos theory, and Discrete Wavelet Transform (DWT). Using confusion and diffusion processes, chaos theory is used to modify image pixels. In contrast, bit-plane extraction and DWT are employed to reduce the processing time required for encryption. Multiple cyberattack analysis, including noise and cropping attacks, are performed by adding random noise to the ciphertext image in order to determine the proposed encryption scheme’s resistance to such attacks. In addition, a variety of statistical security analyses, including entropy, contrast, energy, correlation, peak signal-to-noise ratio (PSNR), and mean square error (MSE), are performed to evaluate the security of the proposed encryption system. Moreover, a comparison is made between the statistical security analysis of the proposed encryption scheme and the existing work to demonstrate that the suggested encryption scheme is better to the existing ones.

## 1 Introduction

UAVs are popularly known as drones can be used in real-time applications such as security, communication, transfer of payload, and rescue operations. Drones help us to communicate with other parties where it is difficult to access for humans. Initially, UAVs were used independently, but nowadays, they are integrated with other UAVs to communicate with each other [1–3]. During transmission of data among the UAVs, the attacker can also launch their drone to steal the information which is transmitted between the authentic UAVs or from the UAVs to the ground station.

**Fig 1:**
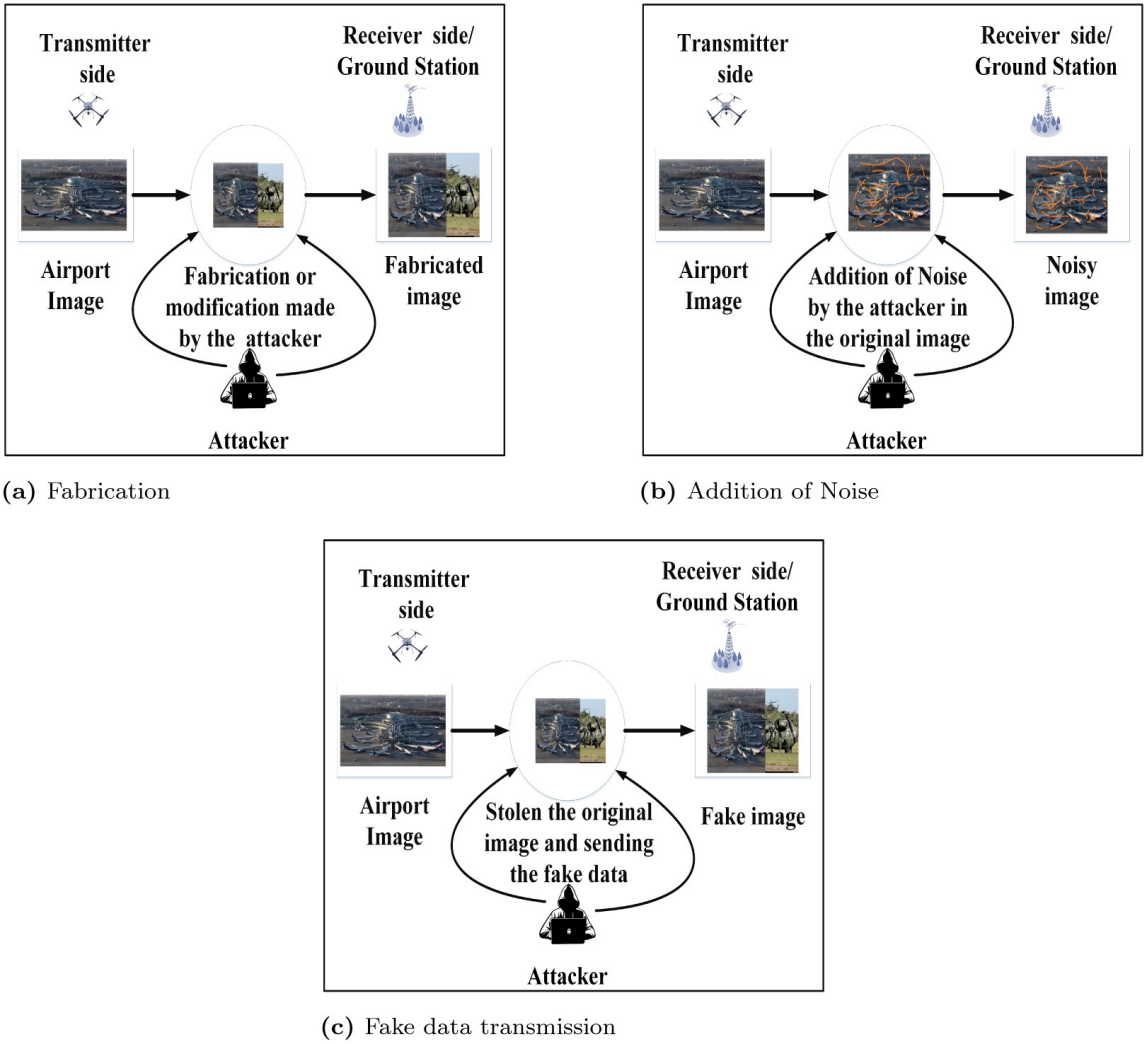
Different categories of attacks. (a) Fabrication, (b) Addition of Noise and (c) Fake data transmission.

UAVs mostly capture the data in the form of images [4]. As in the UAVs, the storage is limited, therefore it is not possible to store multiple images in its memory at a time. To avoid such issues, the UAV must have to send the taken photographs and delete them instantly so that the UAV will have enough storage to save the upcoming data [5–7]. While sending the data without any security protocols, the data can be attacked by the eavesdropper in the following ways:

- Data fabrication
- Addition of noise
- The attacker can send fake data after stealing the original version. Fig (1a)–(1c) shows the pictorial representation of aforementioned attacks.

The data can easily be interrupted or modified if it is transmitted over an insecure channel (Internet) which posses a great security threat [8–12]. Therefore, it is meaning full to encrypt the digital data (images) before sending it to the destination. To secure such data, well-known encryption algorithms such as Advanced Encryption Standard (AES), Data Encryption Standard (DES) and blow fish are proposed in recent years [13–15]. Although such algorithms offers suitable security to the digital images, but due to the number of encryption rounds, the encryption computational time of such techniques is high which is not suitable for real-time applications. However,the encryption proposed in [16–18] can be considered for mobile applications as their performance in terms of computational speed is suitable for such applications.

To reduce time complexity, images can be encrypted using chaos and transformation techniques such as Discrete Cosine transform (DCT) and DWT [19–28]. In the past few years, chaos has played a vital role in image encryption due to its some unique features such as sensitivity in initial conditions, unpredictability, pseudo randomness, ergodicity, and non-deterministic behavior [29–34]. Chaotic systems can be divided into low-dimensional and high-dimensional maps. In low-dimensional chaotic maps such as chaotic sine map and chaotic logistic map consists of less number of initial conditions and control parameters [35, 36]. This makes them easy to implement with fewer resources. On the other side, high-dimensional chaotic maps such as hyper chaotic map and Lorenz system chaos consist of a significant number of initial and control parameters [37, 38]. Although the structure of the high-dimensional chaotic maps is complex, but it occupies more storage and also difficult to implement, requiring more resources and high computational time. Therefore, such high-dimensional chaotic maps are not suitable for real-time applications. The issues of high computational time cannot be ignored in UAVs application as it utilizes limited resources such as finite battery capacity and limited storage that does not allow to store the large data. Storing large data may reduce the computational power of the system [39, 40].

To overcome computational time complexity issues and to provide a suitable security to the digital images, a new technique is proposed based on DWt and chaos theory. The major contributions of the proposed work are as follows:

- A new image encryption scheme is proposed which is especially designed for such real time applications where less processing time is required.
- A DWT is used to decompose the plaintext image at the 5^th^ level in order to minimize the computing time required for encryption. When DWT is used to convert a plaintext image into frequency sub-bands, the sub-bands are reduced by a factor of two. The size of the frequency sub-band at the *nth* level of decomposition will be $\frac{\text{size}\;\text{of}\;\text{image}}{2^{n}}$. Where *n* represents the level of decomposition, Therefore, the sub-band size at the 5^th^ level decomposition will be $\frac{\text{size}\;\text{of}\;\text{image}}{2^{5}}$.
- To enhance the security of the proposed encryption scheme, a new algorithm for the generation of noisy images is presented in order to create the diffusion in the plaintext image.
- The cropping and noise attack are carried out by utilising XOR peration to inject random noise into the ciphertext image.
- Several statistical security measures, including entropy, contrast, energy, mean square error, and peak signal-to-noise ratio are conducted to demonstrate the resilience of the proposed encryption scheme

The rest of the paper is structured as follows: The section 1 is devoted to related work and flaws in existing work. In section 3, bit-plane extraction methodology is explained with an example in which an image portion of size 3 × 3 is taken from the original image. The overview and the detailed steps of the proposed methodology are explained in sections 4 and 5 respectively. In section 6, the experimental results and analysis of the proposed and existing encryption schemes are presented to show the effectiveness of the proposed encryption scheme over the existing work. In the last, section 7 concluded the proposed work.

## 2 Related work

To sort out the issues of computation complexity and providing a high level of security to the digital data, significant research has been carried out in which some of them are based on frequency transformation, and chaotic systems based integrated with substitution boxes (S-boxes) [41–49].

### 2.1 Encryption using transform techniques

Pixels do not undergo any direct manipulation in encryption systems that are based on frequency transforms. It does this by converting the pixels into their frequency components, which may then be utilised in conjunction with the Discrete Cosine Transform (DCT), Discrete Fourier Transform (DFT), and Discrete Wavelet Transform (DWT).

Joshi et al. [50] introduced an image encryption technique in their research work. This scheme is intended to protect digital images from any kind of cyberattack. The authors made use of DWT, which involves first converting the plaintext image pixels into its frequency components, such as LL, LH, HL, and HH frequency sub-bands. The LL frequency sub-band stores the majority of the plaintext information, while the other three sub-bands store the finer details. The authors believed that any of these frequency sub-bands may be used effectively to encrypt the plaintext image. As a direct consequence of this, the amount of time required for the calculation is greatly increased. Therefore, when a low processing time is required, it is not ideal to manipulate all of the frequency sub-bands; rather, just the low frequency sub-band should be manipulated (LL).

In [51], Ding et al. suggested an encryption scheme based on DWT and a high-dimensional chaotic map. The high-dimensional map has the potential to provide better safety, but it is challenging to deploy. As a result, the cryptosystem that is suggested in [51] is not appropriate for use in real-time applications. On the other hand, in [52], a method of partial encryption is suggested, which makes use of both DWT and low-dimensional chaotic maps. Despite the fact that the authors’ strategy to cut down on the amount of computational time was effective since it made use of a low-dimensional chaotic map, On the other hand, there are two important flaws: (a) compression is employed after encryption, which can cause an increase in the amount of time required for encryption; and (b) the suggested encryption technique is applied to both high-frequency and low-frequency components. Both of these flaws result in an increase in the amount of time required for encryption of the plaintext image.

In [53], Li et al. presented a DWT and chaos-based image encryption scheme. In this technique, the authors encrypted a portion of the low-frequency components of the plaintext image after the image was decomposed into four different frequency sub-bands. Due to the fact that certain sections of the low frequency subbands are encrypted, this technique is lossy in nature. Even if the encrypted version of the plaintext image makes the contents of the plaintext image visible, the original image’s pixels are not an exact replica of the original image. As a consequence of this, the approach might be costly in situations in which everybody needs the exact original information. In [54] Li et al. coupled Lifting Wavelet Transform (LWT) with XOR operation to alter the image pixels in order to provide a high degree of security. The resultant XORed images are subjected to further processing in his suggested methodology’s compression operation, which ultimately results in a reduction in the size of the ciphertext image. The method has the benefit of requiring a minimal amount of bandwidth for the transmission of the ciphertext image due to the relatively tiny size of the encrypted image. The time required to encrypt a digital image with dimensions of 256 × 256 pixels takes around three seconds, which is a duration that is considered quite excessive for use in real-time applications.

### 2.2 Chaos theory and substitution based encryption techniques

Apart from the transform approaches to encrypt digital images, substitution boxes and chaos theory have played a key role in the field of cryptography. Naseer et al. [55] suggested an encryption method based on chaos-based and substitution permutation networks. The permutation and substitution are applied using the permutation box (P-box) and substitution-permutation box respectively. The authors applied the permutation and substitution one by one on the plaintext image having the size of 256 × 256. As a consequence, encryption computational time may increase. All of the mathematical steps in their proposed encryption scheme can be performed simultaneously to reduce the cost of computing.

Encryption methods that are based on chaos also include S-boxes as a key component. It is vital to utilise a robust S-box that is able to withstand any security attacks in order to improve the efficacy of chaos and substitution-based encryption schemes that also make use of S-boxes. This is done in order to make the schemes more secure. Shafique et al. [42] presented a novel approach to construct an S-box, which was based on a 1-dimensional cubic logistic map (CLM). In his work, 256 elements of the S-box have been created with the help of the CLM. This method ensures that the right initial conditions are applied. In order to demonstrate the effectiveness of the suggested S-box, a number of different security evaluations were carried out. These evaluations included the Bit independent criterion (BIC), the Linear Approximation Probability (LP), and the Differential Approximation Probability (DAP).

Using a single S-box in any encryption technique may reduce the strength of the entire encryption process. The vulnerabilities of utilising a single S-box are addressed in [56]. In [56], Anees et al. presented the solution of employing multiple S-boxes in an encryption scheme to increase the security of the encrypted images. The authors employed numerous S-boxes instead of a single S-box. The selection of a particular S-box is dependent on the random sequence created using a chaotic logistic map. The multiple S-boxes based image encryption exhibited much better results when compared with the single S-box results. However, the pattern of the plaintext image may be seen in the encrypted image. To tackle this problem, Ahmad et al. [57] continue with the technique described in [56], and build another multiple S-box based image encryption approach which yields considerably better results.

Hua et al. introduced a novel encryption method based on a 2D Sine logistic modular map (SLMM), which is comprised of chaotic sine and logistic maps. [58]. Although both of these chaotic maps exhibit highly non-linear behaviour, it is possible to breach the security of the proposed method by using chaotic signal estimating technologies [59]. Sahteesh et al. [60] employed S-P networks and chaotic maps in their research, which included the use of pixel permutation and diffusion. However, the only XOR operation was used for diffusion purposes, which degrades the security of their proposed algorithm. Liu et al. [61] proposed a new encryption scheme that improved the S-box-based schemes by adding noise and converting the data into blocks. This scheme worked well for images that contained a greater number of grey levels, but it was unable to successfully encrypt images that contained a lower number of grey levels.

The overview of the existing encryption schemes is presented in Table 1 in which several features such as category, advantages, drawbacks and their related possible countermeasures are displayed.

**Table 1:** Summary of transform based existing encryption schemes.

Category	Technique	Advantages	Vulnerabilities	Countermeasures
**Frequency transform based encryption**	DWT based encryption [50]	Can resist brute force attack due to large key space	Time inefficient	Encrypt only low frequency component instead of all frequency components including low and high frequencies
	DWT and chaos-based encryption [51]	Strong security	Difficult to implement	Use low dimensional chaotic map
	Partial image encryption [52]	Suitable level of security	Compression followed by encryption makes the encryption scheme time inefficient	Encryption without compression
	DWT and chaos-based image encryption scheme [53]	Low processing time	Data loss during decryption process	Used full image encryption instead of partial or selective image encryption
	Lifting Wavelet Transform (LWT) based encryption [54]	Low bandwidth required	Non-resistive against chosen plaintext and ciphertext attack	Multiple rounds of encryption may use to withstand cyberattcks
**Chaos and substitution based encryption techniques**	substitution permutation networks-based encryption [55]	Resistive against cyber attacks such as entropy attack, chosen plaintext and ciphertext attacks	Time inefficient	Use robust substitution permutation processes
	Chaos-based S-box is proposed [42]	Easy to implement	-	-
	Multiple S-boxes [56]	Can replace single S-box with multiple boxes	Patterns of plaintext image cannot be encrypted properly	Permutation may apply before using multiple Boxes
	Multiple S-boxes [57]	Properly encrypted plaintext image patterns	Time inefficient	Perform multiple operations simultaneously
	S-P networks and chaos based image encryption [60]	Weak security	A bit fast algorithm	Replace XOR operation with some powerful transformation operation
	Chaos based [61]	Improved S-box based schemes	Cannot properly encrypt those images which contain less number of gray levels	Use S-boxes and integrate with chaotic maps

Keeping in mind the issues discussed in this section, in this paper, an encryption scheme is proposed which is time efficient and is capable of providing a significant level of security to the digital images. The proposed encryption scheme is presented specifically for such real time applications in which less processing time is required to encrypt the information. For reducing the computational complexity of the encryption scheme, a DWT is used to decompose the plaintext image at 5^th^ level decomposition. At the 5^th^ level decomposition, the size of the plaintext image becomes $\frac{M}{2^{5}}$ × $\frac{N}{2^{5}}$. Therefore, at that level, fast encryption may be performed. Moreover, to further reduce the computational complexity of the encryption algorithm, only low-frequency sub-bands are considered.

## 3 Bit plane extraction

**Fig 2:**
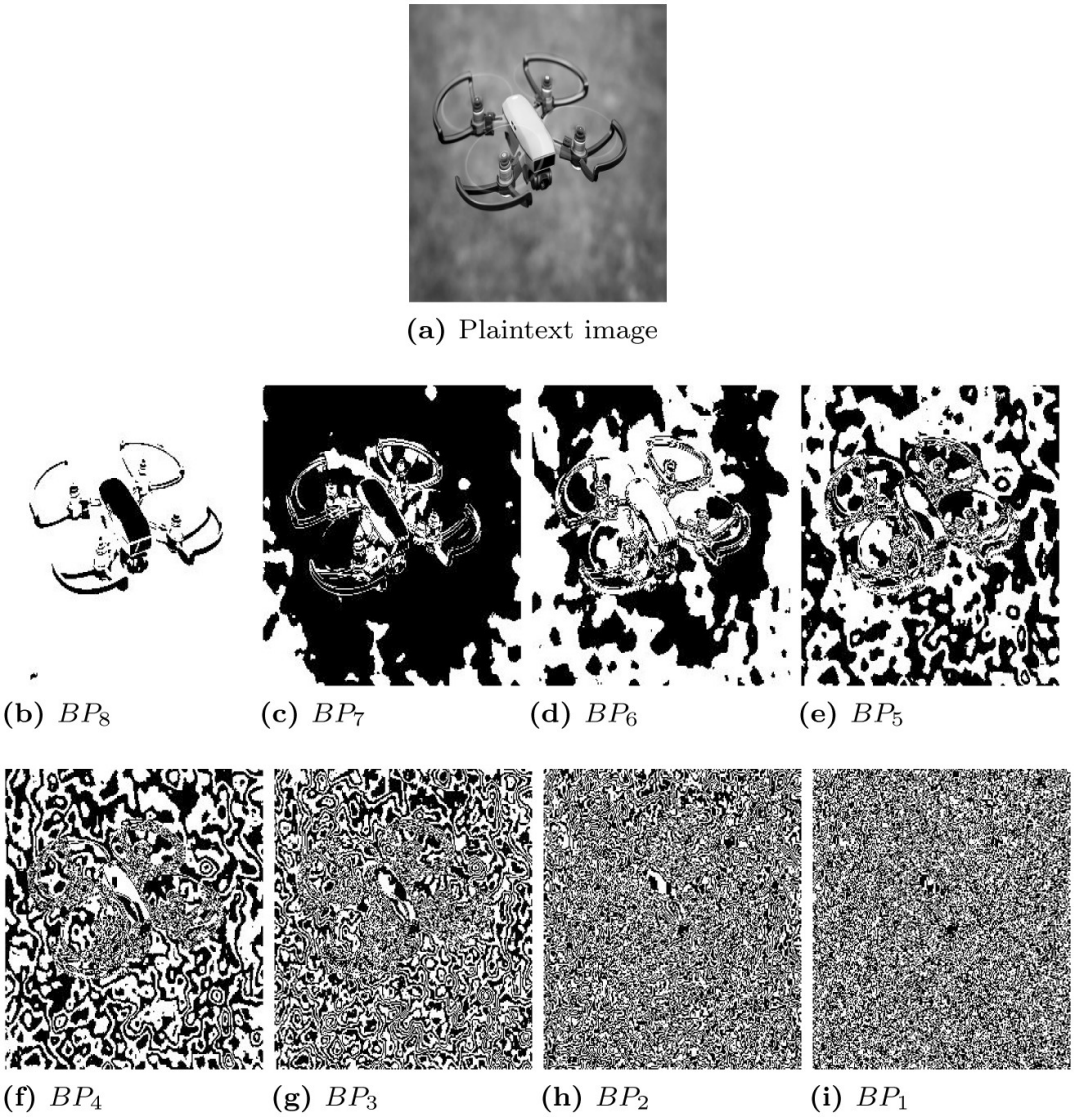
Pliantext image and its corresponding extracted bit-planes. (a) Plaintext image, (b) *BP*_8_, (c) *BP*_7_, (d) *BP*_6_, (e) *BP*_5_, (f) *BP*_4_, (g) *BP*_3_, (h) *BP*_2_, and (i) *BP*_1_.

For the eight-bit plaintext image, eight bits can be extracted using the Eq (3). Each bit-plane consists of a different amount of plaintext image information. The four least significant bit planes (*BP*_1_, *BP*_2_, *BP*_3_, and *BP*_4_) have the least amount of information, as shown in Fig 2. The percentage of information in each bit-plane can be calculated using Eq (2) and the statistical values of information percentage in each bit-plane are displayed in Table 2.

$$\begin{array}{c} BP_{1}=\left(\frac{I}{1}\right)mod2,\;BP_{2}=\left(\frac{I}{2}\right)mod2\;BP_{3}=\left(\frac{I}{4}\right)mod2,\;BP_{4}=\left(\frac{I}{8}\right)mod2\\ BP_{5}=\left(\frac{I}{16}\right)mod2,\;BP_{6}=\left(\frac{I}{32}\right)mod2\;BP_{7}=\left(\frac{I}{64}\right)mod2,\;BP_{8}=\left(\frac{I}{128}\right)mod2 \end{array}\}$$ (1)

$$\begin{array}{c} I_{i}=\frac{2^{i-1}}{\sum_{i=1}^{8}2^{i-1}} \end{array}$$ (2)

**Table 2:** Information percentage.

*BP*_*i*_ (i = 1, 2, …8)	Information percentage
1	0.30
2	0.79
3	1.42
4	3.12
5	6.25
6	12.23
7	25.7
8	50.20

From Table 2, it can be seen that most of the plaintext information lies in the most significant bit planes (*BP*_8_ − *BP*_5_) and therefore, only such bit-planes are under consideration for the encryption of plaintext image to reduce the encryption computational time.

Apart from the consideration of only most significant bit planes, the simultaneous process of confusion (which refers to scrambling) and diffusion (which refers to the change in the pixel values) also play a vital role to reduces the overall encryption computational time. The process of achieving confusion and diffusion simultaneously is explained in Example 1.

**Example 1**: Let we have an image I is:

$$I=\left[\begin{array}{ccc} 120 & 110 & 96\\ 200 & 186 & 116\\ 174 & 55 & 169 \end{array}\right]$$

Convert the pixel values of image I into binary values:

$$\left[\begin{array}{ccc} 01111000 & 01101110 & 01100000\\ 11001000 & 10111010 & 01110100\\ 10101110 & 00110111 & 10101001 \end{array}\right]$$

For the extraction of first binary bit-plane (*BP*_1_ bit-plane), *LSB*_1_ of all the pixel values will be considered, and for the extraction of second binary bit-plane (*BP*_2_ bit-plane), *LSB*_2_ of all the pixel values will be considered and so on. The eight extracted binary bit-planes from image *I* will be:

$$BP_{8}=\left[\begin{array}{ccc} 0 & 0 & 0\\ 1 & 1 & 0\\ 1 & 0 & 1 \end{array}\right],BP_{7}=\left[\begin{array}{ccc} 1 & 1 & 1\\ 1 & 0 & 1\\ 0 & 0 & 0 \end{array}\right],BP_{6}=\left[\begin{array}{ccc} 1 & 1 & 1\\ 0 & 1 & 1\\ 1 & 1 & 1 \end{array}\right],BP_{5}=\left[\begin{array}{ccc} 1 & 0 & 0\\ 0 & 1 & 1\\ 0 & 1 & 0 \end{array}\right]$$

$$BP_{4}=\left[\begin{array}{ccc} 1 & 1 & 0\\ 1 & 1 & 0\\ 1 & 0 & 1 \end{array}\right],BP_{3}=\left[\begin{array}{ccc} 0 & 1 & 0\\ 0 & 0 & 1\\ 1 & 1 & 0 \end{array}\right],BP_{2}=\left[\begin{array}{ccc} 0 & 1 & 0\\ 1 & 0 & 1\\ 1 & 1 & 0 \end{array}\right],BP_{1}=\left[\begin{array}{ccc} 0 & 0 & 0\\ 0 & 0 & 0\\ 0 & 1 & 1 \end{array}\right]$$

After the scrambling process, combine the binary values which are placed at (1,1) in each scrambled bit-plane. It will return the eight bits of the first pixel value. similarly, for the second pixel value, combine the binary bits which are placed at (1,2) and so on. The resultant image (*I*′) will be:

$$I^{\prime }=[\begin{array}{ccc} 01101111 & 00010000 & 11101110\\ 10110110 & 01100101 & 10101010\\ 10111000 & 01101010 & 11111000 \end{array}]\to [\begin{array}{ccc} 111 & 16 & 238\\ 182 & 101 & 170\\ 184 & 106 & 248 \end{array}]$$

It can be seen from the image *I*′, the pixel values are completely different from the original image *I* pixels.

## 4 Overview of the proposed encryption scheme

In this scheme, a plaintext image is decomposed into different frequency bands using DWT. To make the proposed scheme time-efficient, a fifth-level DWT decomposition is performed, and only low-frequency components are taken to be dealt with. Apart from the DWT, multiple chaotic maps and the bit-plane extraction method are also used. In the bit-plane extraction method, confusion and diffusion are achieved simultaneously by performing only scrambling operation on extracted bit-planes, which helps to reduce the computational complexity.

The Haar wavelet transform can be represented as *Q*′ = *HPH*^*T*^ in which *P* is a plaintext image having equal number of pixel rows and columns i.e the size of image *P* is *R*(rows) × *R*(columns), *H* represents the Haar transform matrix having the size equal to the plaintext image and *Q* is the transform matrix which contain the Haar basis function *h*_*a*_(*w*). Where *w* ∈ [0 1] and *a* is defined as *a*∣ *a* ∈ *N* ∧ 0 ≤ *a* ≤ *R* − 1}. It can be decompose uniquely as:

### 4.1 DWT and its role in the proposed scheme

**Fig 3:**
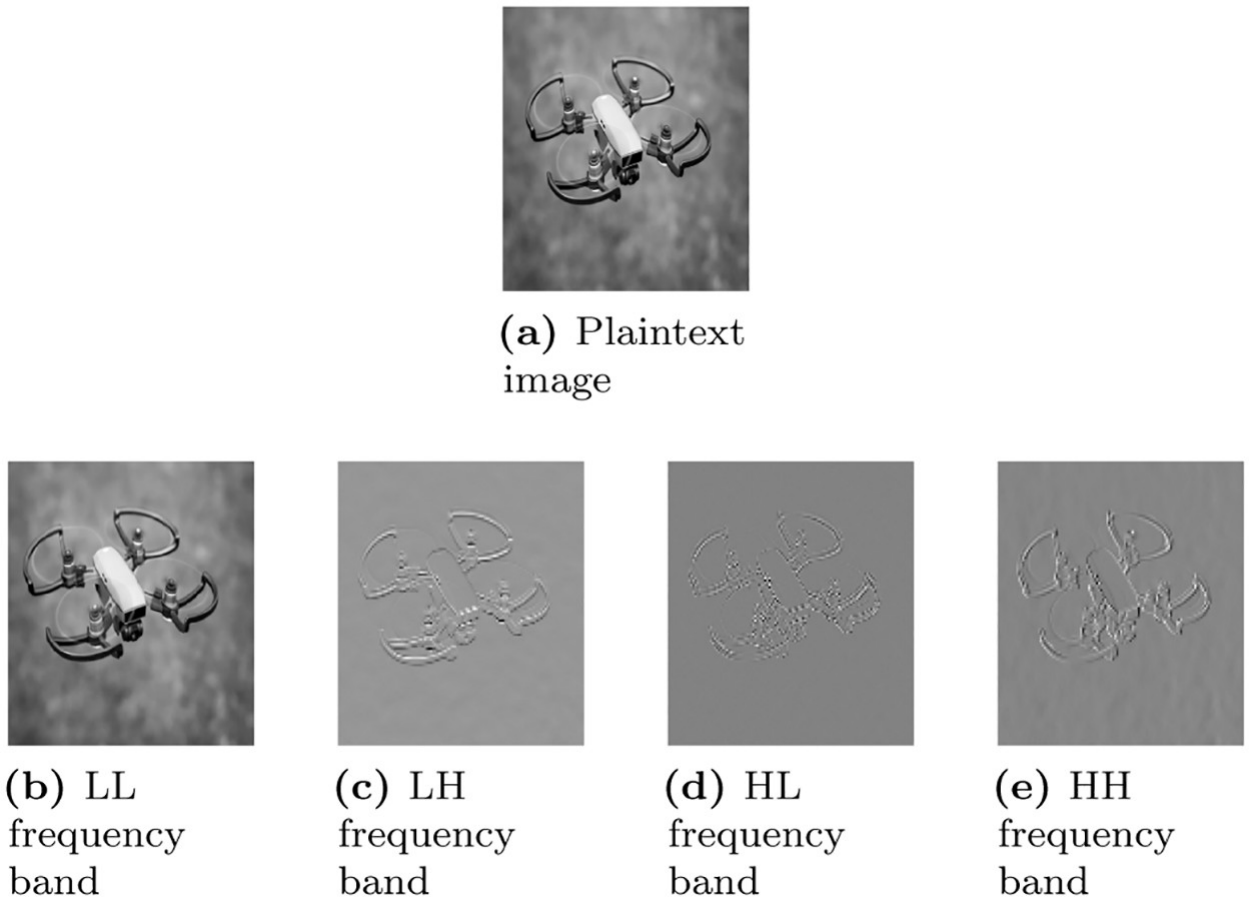
Frequency bands extracted from the drone image using DWT. (a) Plaintext image, (b) LL frequency band, (c) LH frequency band, (d) HL frequency band, and (e) HH frequency band.

DWT is used to decompose the plaintext image into different frequency components. In every iteration of DWT, the plaintext image divides not four frequency sub-bands such as *LL* sub-band, *LH* sub-band, *HL* sub-band, and *HH* sub-band. *LL* sub-bands correspond to the low-frequency components in which more than 90% of the plaintext information is present as shown in Fig 3. Therefore, to reduce the encryption time, only *LL* sub-band should consider for the encryption of plaintext image.

In the proposed research, Haar wavelet is used which can be represented as *W* = *HOH*^*p*^ in whcih *O* is an original image, *H* shows the haar transform array in which the number of rows (*R*) and columns (*C*) are equal to the *R* and *C* of the original image and *T* represents the function of Haar basis *Y*_*x*_(*w*). Where *w* ∈ [0 1] and *x* is defined as *x*∣ *x* ∈ *N* ∧ 0 ≤ *x* ≤ *R* − 1}. It can be split uniquely using Eq (3) [62].

$$\begin{array}{c} Q=2^{f}+L, \end{array}$$ (3)

Where *f* shows the maximum exponential number of 2 and *L* represents the reminder (L = 2^f^ − *Q*). Mathematically, Haar basis function can be defined as:

$$\begin{array}{c} Y_{x}\left(w\right)=\frac{1}{\sqrt{R}}\{\begin{array}{cc} 1 & \;ifx=0\;\&\;0\leq w<1\\ 2^{f/2} & \;ifx>0\;\&\;L/2^{f}\leq w<\frac{L+0.5}{2^{f}}\\ -2^{f/2} & \;ifx>0\;\&\;\left(L+0.5\right)/2^{f}\leq w<\frac{L+1}{2^{f}}\\ 0 & \text{Otherwise} \end{array} \end{array}$$ (4)

**Fig 4:**
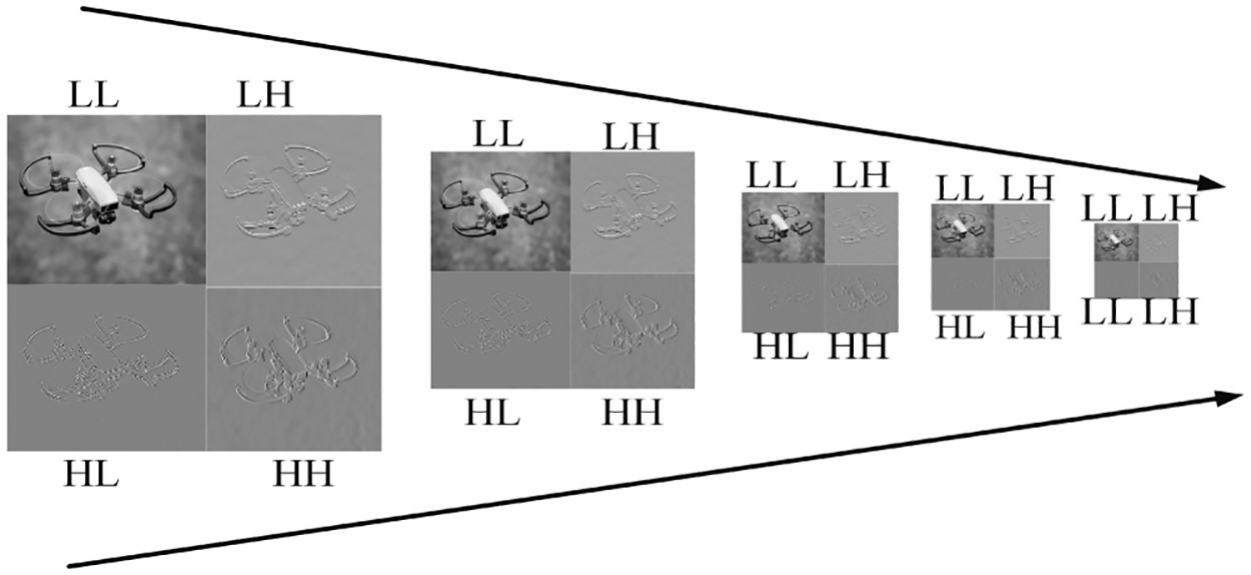
Fifth level DWT decomposition.

After taking the Haar wavelet transform, the size of each frequency sub-band becomes half of the plaintext image. For instance, if the size of the plaintext image is *M* × *N*, the size of each sub-band will be one-half of the plaintext image i.e. $\frac{M}{2}$ × $\frac{N}{2}$. During the second level decomposition, the dimensions of the sub-bands will be $\frac{M}{4}$ × $\frac{N}{4}$ and so on. The decomposition level can be changed according to the application. In the proposed encryption algorithm, fifth-level DWT decomposition is performed to reduce the encryption computational time. Fig 4 shows the fifth level DWT decomposition.

### 4.2 Chaotic maps used in the proposed scheme

The proposed scheme is also based on chaotic maps. In the proposed work, the chaotic logistic and chaotic sine maps are used for generating the random sequqences and random image to create the diffusion in the plaintext image. The details of such chaotic aps can be seen in [63, 64].

Over the last few years. Chaotic maps are widely used in image encryption due to their tremendous properties such as sensitivity to initial conditions, ability to generate randomness and non-periodicity. Following are the chaotic maps used in the proposed work:

#### 4.2.1 Chaotic logistic map (CLM)

CLM is a one-dimensional chaotic map that is used to generate a random sequence for the permutation of rows of pixels in the plaintext image. The reason for choosing the one-dimensional chaotic map over the high-dimensional maps is easy to implement and comparatively faster. Mathematically, the chaotic map can be written as:

$$\begin{array}{c} \Omega_{n+1}=\lambda \times \Omega \left(1-\Omega_{n}\right) \end{array}$$ (5)

Where Ω_0_ and λ are the initial condition and the control parameters respectively. The ranges of these parameters are:

Ω∈(0,1)

λ∈(0,4)

**Fig 5:**
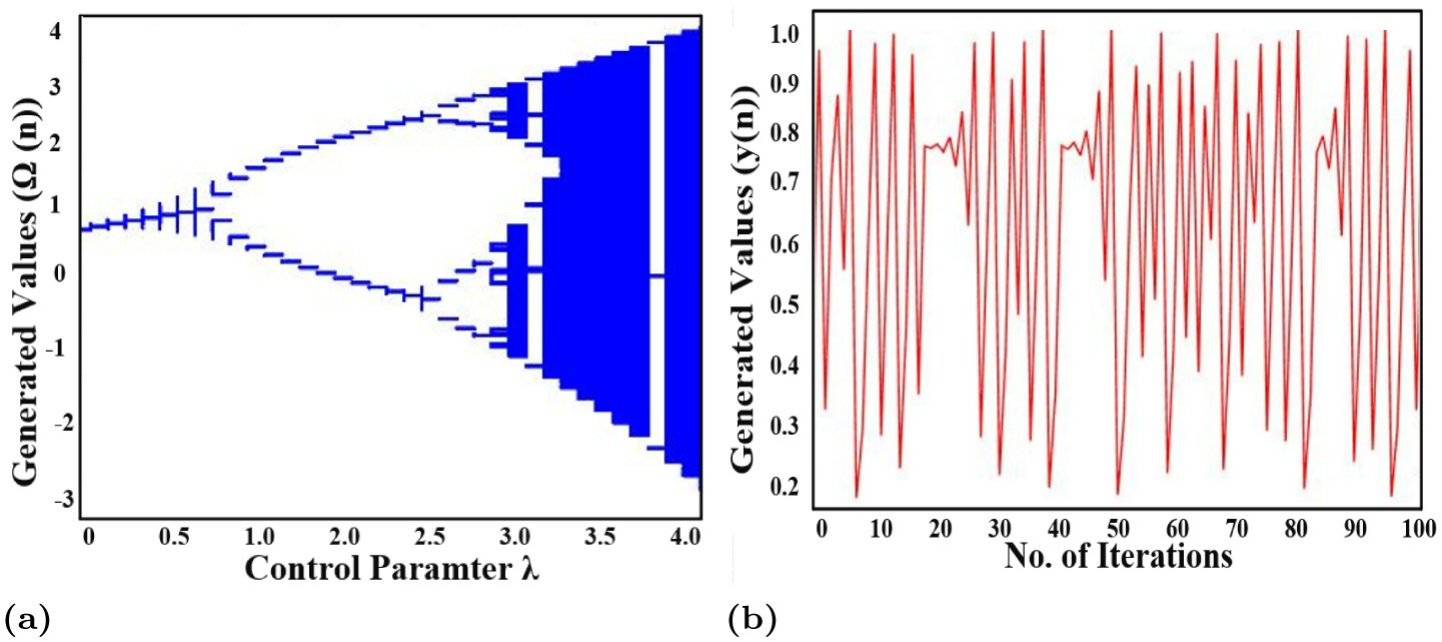
(a) Bifurcation diagram of CLM and (b) random sequence generated using CLM.

To generate the random sequence using CLM, Ω must lie in the chaotic range. Fig 5(a) shows the bifurcation diagram of the logistic map in which it can be seen that the CLM shows chaotic behavior when the value of Ω lies in the range [0, 4]. Moreover, the sequence generated using CLM when the value of λ is selected from the chaotic range is shown in Fig 5(b) in which a lot of random values can be seen graphically.

#### 4.2.2 Chaotic sine map (CSM)

Like CLM, CSM is a one-dimensional chaotic map. CSM is used in the proposed work to generate the random vector for the permutations of pixels columns in the plaintext image. CTM is given in Eq (6).

$$\begin{array}{c} y_{n+1}=\beta *sin\left(\pi \alpha_{i}\right) \end{array}$$ (6)

**Fig 6:**
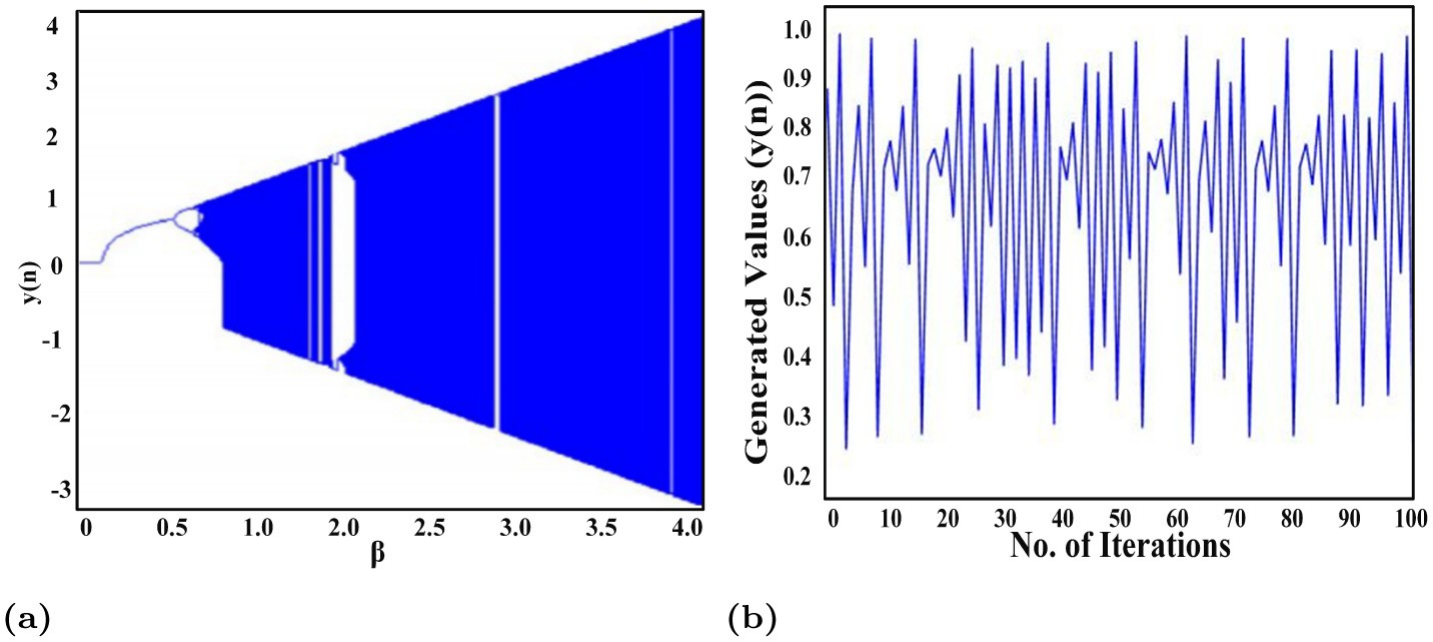
(a) Bifurcation diagram of CSM and (b) Random sequence generated using CTM.

Where *y*(0) and *β* are the initial or seed values which lie in the range [0, 1] and [0.87, 1] respectively. The chaotic range for CSM is [0.87, 1]. As it can be seen in Fig 6(a), most of the different values are generated in their chaotic range. Therefore, to generate the maximum random values, the value of *α* is selected as 0.91. This effect is shown in Fig 6(b).

## 5 DWT based proposed encryption scheme

The three major components of the proposed encryption are bit-plane extraction, chaotic maps (logistic and sine map) and DWT. The secret keys which are used in the proposed work are generated using chaotic maps. The process of key generation is given in section 5.1.

### 5.1 Key generation process

1. Iterate Eqs 5 and 6 *M* × *N* times using the initial conditions *ω*, λ, *β* and *y* to generate different random values. Such random values are stored in an array known as *key* − *stream*(*KS*).
2. The values generated in *KS* are in the range (0 1). Therefore, to amplify the *KS* values, any large multiplication factor (*N*) is used.
3. The amplified values are converted into an integers values by truncating all the numbers which are placed after the decimal point.
4. The values generated in step 3 are now restricted in the range [0 255] using the modulo operation as follows:
  $$\begin{array}{c} X=uint8(mod(floor\left(\left(RN_{1}\right)*N_{1}\right);\;\text{Keys}\;\text{are:}\;\Omega ,\lambda \end{array}$$ (7)
  $$\begin{array}{c} Y=uint8(mod(floor\left(\left(RN_{1}\right)*N_{2}\right);\;\text{Keys}\;\text{are:}\;\beta ,y_{0} \end{array}$$ (8)
5. The sequences *X*, and *Y* are used as scrambling keys for the permutation of rows and columns of the bit-planes (*BP*_8_ and *BP*_7_).

### 5.2 Encryption procedure

**Fig 7:**
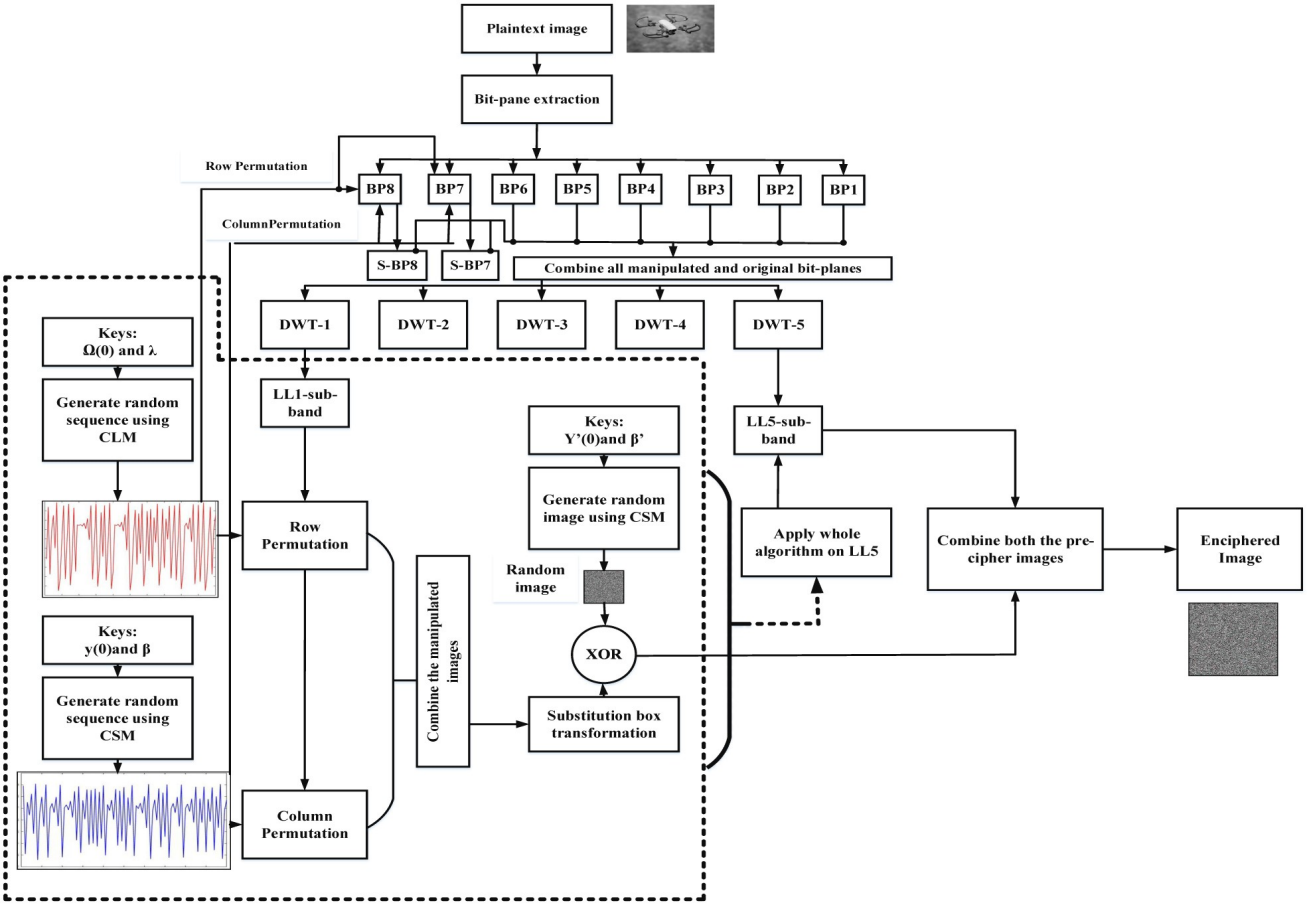
Proposed algorithm for fast image encryption.

The image encryption scheme proposed in this paper is for-real time applications. Step wise block diagram of the proposed encryption model is show in Fig 7. While the detail of each step the proposed scheme is given below:

- Take a plaintext image of sized *M* × *N* and decompose into eight bit-planes. For the encryption procedure, only *BP*_8_ and *BP*_7_ are considered because more than 90% of plaintext information is present in such bit-planes. Therefore, it is not necessary to encrypt all the bit-planes.
- For row and column scrambling, the sequences generated *X*, *Y* and *Z* in the key generation process are used.
- Combine the permuted bit-planes (*S* − *BP*_8_ and *S* − *BP*_7_) and the unchanged bit-planes using Eq (9).
  $$I^{\prime }=2\times (2\times (2\times \left(2\times \left(2\times \left(2\times \left(2\times S-BP_{8}+S-BP_{7}\right)+BP_{6}\right)+BP_{5}\right)+BP_{4}\right)+BP_{3})+BP_{2})+BP_{1}$$ (9)
- Apply DWt to *I*′ up to 5^th^ level. The size of the *LL*_5_ will be $\frac{M}{2^{5}}$ × $\frac{N}{2^{5}}$. For the encryption time reduction, only *LL* sub-bands will be considered because most of the information of the plaintext image present in the low-frequency sub-bands. The extracted frequency sub-bands are shown in Eqs 10–13.
  $$\begin{array}{c} DWT_{1}\to \;\left[LL_{1},LH_{1},HL_{1},HH_{1}\right], \end{array}$$ (10)
  $$\begin{array}{c} DWT_{2}\to \;\left[LL_{2},LH_{2},HL_{2},HH_{2}\right], \end{array}$$ (11)
  $$\begin{array}{c} DWT_{3}\to \;\left[LL_{3},LH_{3},HL_{3},HH_{3}\right], \end{array}$$ (12)
  $$\begin{array}{c} DWT_{4}\to \;\left[LL_{4},LH_{4},HL_{4},HH_{4}\right] \end{array}$$ (13)
  $$\begin{array}{c} DWT_{5}\to \;\left[LL_{5},LH_{5},HL_{5},HH_{5}\right] \end{array}$$ (14)
- For the rows and column permutation of *LL*_5_ frequency band, the sequences *X* and *Y* are used.
- Combine both the manipulated images generated in the above two steps and apply the S-box transformation on them to get substituted image (*S*_*image*_). The detailed process of S-box transformation is given in [56].
- To create diffusion in the ciphertext image, *XOR* is applied on the *S*_*image*_ and the random image. The random image is generated according to algorithm 1.

**Algorithm 1** Substitution of *LL*_4_ sub-band values with S-box

**Start** Input: Initial conditions as secrete keys (λ and Ω_0_), CLM.

→ Implementation of CLM to generate *M* × *N* values.

→ Stored the generated values in an array *ϕ*.

→ Update *ϕ* using the below Equation:

$$\begin{array}{c} \theta =\phi_{i}*K_{num} \end{array}$$ (15)

 → *K*_*num*_ is a key num which is used to amplify the values stored in *ϕ*.

→ Apply floor function to truncate the fractional digits and stored the result in *α*.

→ To restrict the generated value in the range [0 255], apply modulo function as given below:

$$\begin{array}{c} M=modulo(\alpha ,256) \end{array}$$ (16)

→ Convert *M* into 2-D array (*R*_*image*_).

**End**

**Fig 8:**
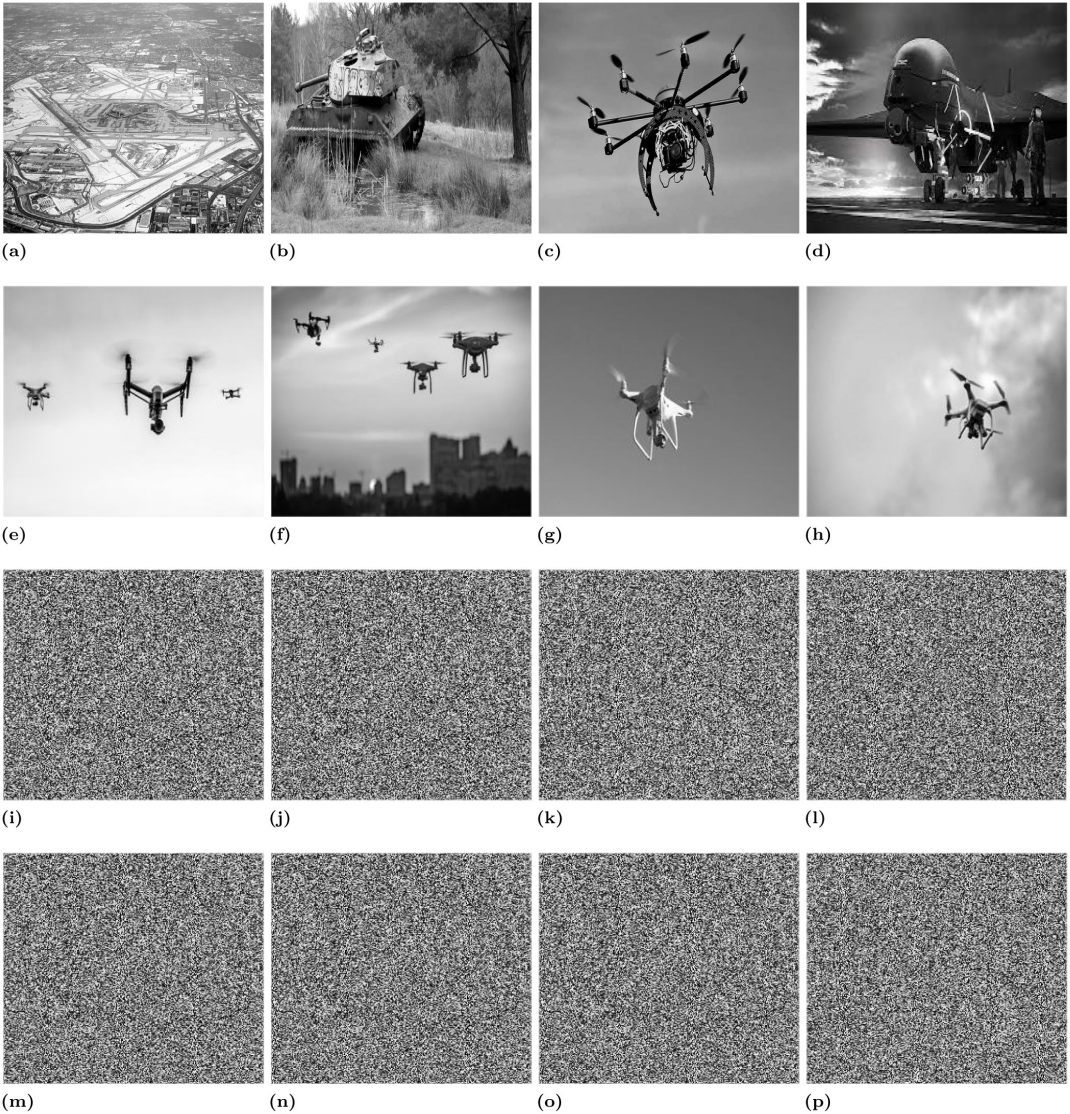
Plaintext and their corresponding enciphered images.

**Fig 9:**
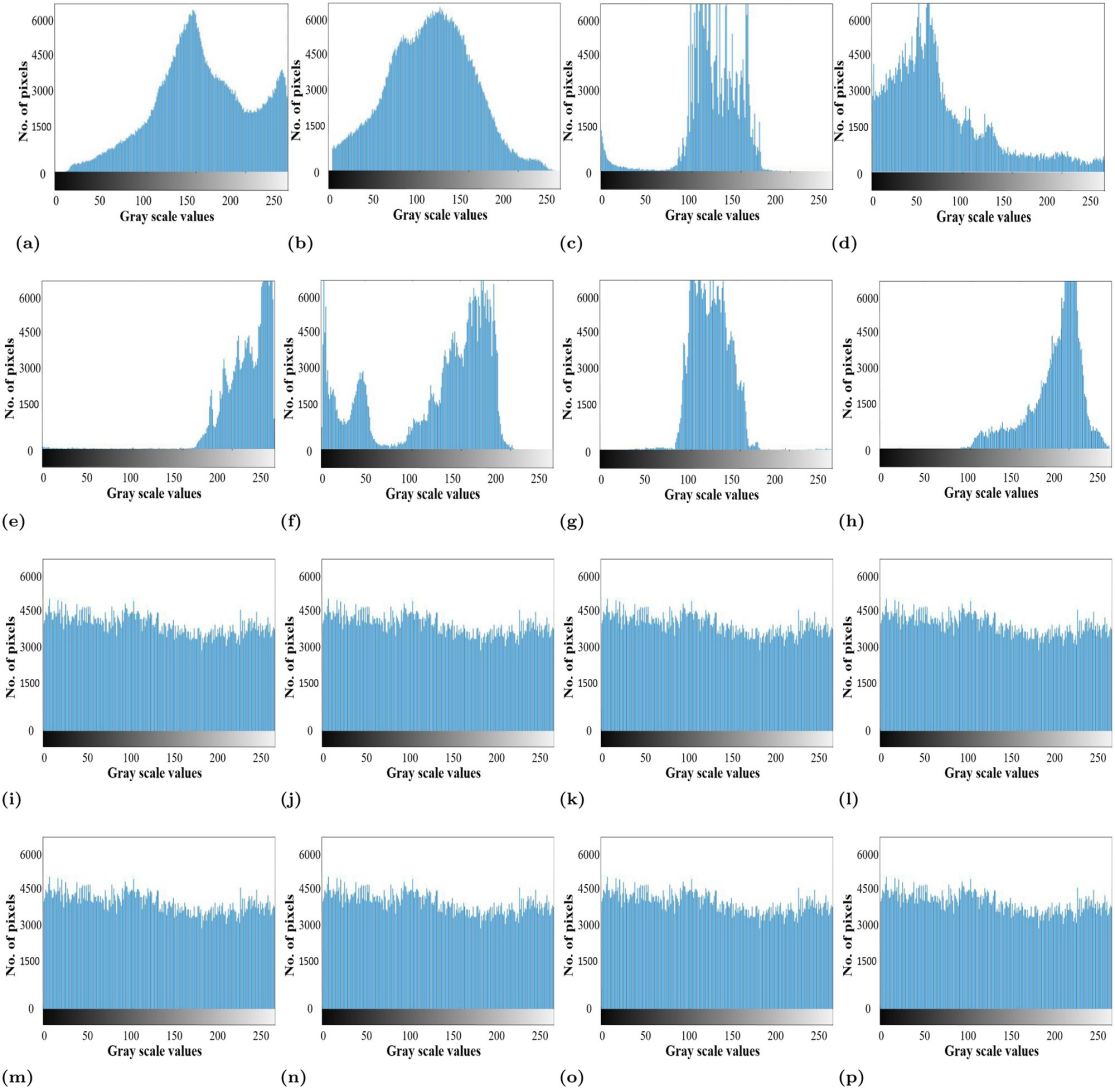
Histograms of plaintext images and the histogram of the corresponding enciphered images.

- Eq (17) is used to apply *XOR* operation on the *S*_*image*_ and *R*_*image*_ to get final encrypted image.
  $$\begin{array}{c} \text{Encrpted}\;\text{image:}\;C^{{}^{\prime }}=\sum_{i=1}^{M}\sum_{j=1}^{N}R_{image}\left(i,j\right)⊕\sum_{i=1}^{M}\sum_{j=1}^{N}S_{image}\left(i,j\right) \end{array}$$ (17)
  The plaintext images and their corresponding encriphered images which are encrypted using the proposed encryption scheme are shown in Fig 8. While the histograms of the plaintext and enciphered images are shown in Fig 9.

The two advantages of this scheme are: the computational complexity of the proposed encryption algorithm is low, and the image recovered in its decryption holds the original pixel values. Therefore, it is a lossless encryption scheme.

## 6 Security analysis

To gauge the proposed encryption scheme, several statistical security analyses such as entropy, correlation contrast, peak signal to noise ratio, and mean square error. Moreover, apart from the statistical analyses, several attacks such as cropping attacks, noise addition attack and brute force attack are also performed to evaluate the robustness of the proposed encryption.

### 6.1 Histogram analysis

A histogram of an image shows the pixel distribution. The histogram analysis is used to evaluate the performance of the encryption scheme. For the strong encryption scheme, the histogram of the ciphertext image should be flat, uniform, and completely different from the histogram of the plaintext image. The histogram of the plaintext and its corresponding images is shown in Fig 8 where it can be seen that the pixel distribution in the histogram of the plaintext images is fairly uniform, which makes them different from the histogram of the plaintext images. Moreover, the uniformity in the pixel distribution reveals that the proposed encryption scheme can resist the histogram attack.

### 6.2 Histogram variance analysis

Variance is another parameter to evaluate the uniformity of the histogram of the ciphertext images. This metric may be considered more reliable because it gives the statistical values rather than the histogram visualization. Variance can be calculated as [65]:

$$\begin{array}{c} Var\left(P\right)=\frac{1}{256}\sum_{L=1}^{256}{\left[p_{i}-E\left(P\right)\right]}^{2} \end{array}$$ (18)

Where *P* is the pixels stream, *P* = {*p*_1_, *p*_2_, *p*_3_ ….,*p*_256_, *p*_*i*_ is the pixel value at *L*^*th*^ position and $E(P)=\frac{1}{256}\sum_{L=1}^{256}$ *p*_*i*_. For strong encryption, variance values should be low. Table 3 shows the different variance values for the enciphered images generated from the proposed and existing encryption schemes. From the variance values, it can be seen that the proposed scheme performs better than the existing ones.

**Table 3:** Histogram variance analysis.

Plaintext images	Ref [66]	Ref [67]	Ref [68]	Ref [69]	Ref [70]	Ref [71]	Ref [72]	Ref [73]	Proposed
**Airport**	270.671	271.379	269.630	278.136	274.3694	269.679	270.669	268.368	256.637
**Tank**	260.697	261.378	270.672	276.994	260.978	266.330	259.679	270.641	260.300
**Drone**	271.370	271.336	272.039	277.698	276.451	271.036	270.678	273.971	258.633
**Military base**	270.637	271.336	277.987	276.831	270.379	275.689	275.986	275.300	261.596
**Ground station**	270.687	271.678	274.689	271.678	279.678	270.354	271.669	273.067	263.116

### 6.3 Maximum deviation

A cryptographic algorithm’s quality may be determined by the deviation in pixel values between the plaintext and ciphertext images [45]. If the deviation (changes) in pixels between the plaintext and ciphertext images is the maximum, the encryption technique is the more robust in terms of security. Mathematically, maximum deviation can be written as:

$$\begin{array}{c} M_{A}=\frac{A_{0}+A_{N-1}}{2}+\sum_{L=1}^{N-2}A_{L} \end{array}$$ (19)

Where *N*, and *A*_*L*_ represent the total number of gray levels and the amplitude of the difference histogram at index *L* respectively. A higher value of “M” implies that the ciphertext is significantly different from the plaintext image. The results of the maximum deviation for the proposed method and the existing algorithms are shown in Table 4. According to a comparison of average values of maximum deviations, the suggested encryption algorithm, as well as methods in [66, 67], have superior performance than the other comparable schemes. In light of such findings, we may infer that the maximum deviations of the proposed method do not expose any meaningful information regarding the encryption’s quality.

**Table 4:** Maximum deviation.

Plaintext images	Ref [66]	Ref [67]	Ref [68]	Ref [69]	Ref [70]	Ref [71]	Ref [72]	Ref [73]	Proposed
**Airport**	25110	25897	24998	24631	25300	25001	25317	24630	25978
**Tank**	25993	25796	24689	24336	25700	25314	25371	24998	26789
**Drone**	26196	26036	24687	24303	25003	24998	25003	24698	26231
**Military base**	25978	26639	24367	25031	24889	24697	24889	24631	26791
**Ground station**	25830	25136	25003	25013	25112	24983	24320	24316	25590

### 6.4 Irregular deviation

To check the encryption quality, only *M*_*D*_ is not enough. Another metric *I*_*D*_ is used to assess the encryption quality of the enciphered image by determining how close the statistical distribution of deviation between the original and enciphered image is to a uniform statistical distribution. *I*_*D*_ can be calculated as:

$$\begin{array}{c} I_{D}=\sum_{L=0}^{N-1}\left|A_{L}-B_{H}\right| \end{array}$$ (20)

Where *A*_*L*_ is the peak of the histogram at position *L* and *B*_*H*_ is the average sum of the histogram values. The lower the value of *I*_*D*_, the better the enciphered image quality. The values of *I*_*D*_ for the proposed exciting encryption schemes are displayed in Table 5 where it can be seen that the *I*_*D*_ values for the proposed encryption method are lower than the existing ones, which reflects the better strength of the proposed encryption scheme when it compares with other.

**Table 5:** Irregular deviation.

Plaintext images	Ref [66]	Ref [67]	Ref [68]	Ref [69]	Ref [70]	Ref [71]	Ref [72]	Ref [73]	Proposed
**Airport**	46978	47635	48569	47301	46631	47780	47889	46663	45031
**Tank**	45569	46996	47894	46687	49978	47886	48886	46670	45064
**Drone**	46691	47630	47133	49963	48955	47790	49371	46698	45666
**Military base**	46791	46698	47656	46687	47699	46660	46901	47760	45339
**Ground station**	46975	46639	47339	48664	46881	47720	46698	45966	45798

### 6.5 Entropy

Entropy is used to find the robustness in the plaintext or ciphertext image. More randomness in an image results in a higher value of entropy. This relation is shown in Eq (21):

$$\begin{array}{c} Ent\propto randomness \end{array}$$ (21)

Entropy can be calculated as:

$$\begin{array}{c} Entropy=-\sum p\left(k_{i}\right)log_{2}p\left(k_{i}\right) \end{array}$$ (22)

Where: *k*_*i*_ is the probability of occurrence in the variable i.

The maximum entropy value depends on the nature of the image. For instance, if the image is eight-bit, the entropy cannot be exceeded to eight [11]. Similarly, for the binary images, the maximum entropy value can be two. In the proposed scheme, eight-bit images are tested. This means, if the entropy value is close to eight, the proposed scheme can be considered a secure scheme. In Table 6, the entropy values for the different ciphertext images are displayed. Moreover, a comparison with the existing is also shown in Table 6. Where it can be seen that the entropy values for the proposed scheme are much closer to the ideal value which is eight. Also, the entropy values for the existing scheme are less than the entropy values of the proposed encryption scheme.

**Table 6:** Information entropy analysis.

Plaintext images	Ref [66]	Ref [67]	Ref [68]	Ref [69]	Ref [70]	Ref [71]	Ref [72]	Ref [73]	Proposed
**Airport**	7.9871	7.9953	7.9920	7.9935	7.9971	7.9970	7.9963	7.8963	7.9990
**Tank**	7.9765	7.9963	7.9915	7.9991	7.9986	7.9941	7.9453	7.8983	7.9991
**Drone**	7.9796	7.9970	7.9903	7.9943	7.9965	7.9936	7.9516	7.8716	7.9990
**Military base**	7.9899	7.9932	7.9934	7.9968	7.9980	7.9926	7.9695	7.8896	7.9989
**Ground station**	7.9886	7.9975	7.9971	7.9982	7.9971	7.9912	7.9593	7.8640	7.9986

### 6.6 Correlation

Correlation between the image pixel shows the relationship between the intensity of the pixel values. i.e. how close the pixel values are. Greater the difference between the pixel values shows the minimum correlation [74]. Mathematically, it can be written as:

$$\begin{array}{c} Correlation\propto \frac{1}{\text{pixel}\;\text{difference}} \end{array}$$ (23)

Correlation between the image pixel can be calculated as:

$$\begin{array}{c} \text{CorrCoff}=\frac{Cov(w,t)}{\sigma_{w}\sigma_{t}},\;\sigma_{w}=\sqrt{VAR_{w}},\;\;\sigma_{t}=\sqrt{VAR_{t}}\;\;\\ \text{VAR}(\text{n})=\frac{1}{R}\sum_{u=1}^{R}{\left({\text{n}}_{s}-\text{E}(\text{n})\right)}^{2},\;\text{Cov}(\text{n},\text{m})=\frac{1}{R}\sum_{u=1}^{R}\left({\text{n}}_{s}-\text{E}(\text{n})({\text{h}}_{s}-\text{E}(\text{m}\right))) \end{array}$$

Where: E and *σ* represent the expected value operator and the standard deviation respectively.

In the plaintext image, the correlation between the pixel values is always high because the content in the plaintext image can easily be visualized. In contrast, a ciphertext image in which the pixel is properly concealed shows less correlation between the pixels. Therefore, it is always required that the correlation value of pixels in the ciphertext image should be less, so that, no content can be visualized in an encrypted image [75]. Table 7 shows the correlation analysis of the plaintext images, existing schemes, and the proposed scheme. It can be analyzed from Table 7, that the correlation values of the ciphertext images which are generated through the proposed scheme are significantly less than the plaintext images and exiting schemes.

**Table 7:** Correlation analysis.

Plaintext images	Ref [66]	Ref [67]	Ref [68]	Ref [69]	Ref [70]	Ref [71]	Ref [72]	Ref [73]	Proposed
**Airport**	0.0027	0.0015	0.0018	0.0021	0.0015	0.0012	0.006	0.0019	0.0001
**Tank**	0.0019	0.0011	-0.0021	-0.0016	0.0011	0.0022	0.0027	0.0013	-0.0017
**Drone**	0.0023	-0.0021	-0.0015	0.0021	-0.0015	-0.0020	0.0041	-0.0034	-0.0001
**Military base**	0.0027	-0.0035	-0.0023	-0.0023	0.0042	0.0031	0.0015	0.0023	-0.0028
**Ground station**	0.0031	-0.0088	-0.0043	0.0011	0.0035	-0.0043	0.0060	0.0019	-0.0039

### 6.7 Homogeneity

The ability of combinations of pixel brightness results is represented in tabular form by the GLCM. By performing a homogeneity analysis on the distribution in the (GLCM), one can evaluate how close it is to the diagonal of the distribution. The lower the homogeneity measure, the more effective encryption is considered to be. As illustrated in Table 8, the proposed encryption scheme is more effective as compared to the existing schemes. The homogeneity values can be calculated using Eq (24).

$$\begin{array}{c} H_{om}=\sum_{x}\sum_{y}\frac{p(x,y)}{1+|x-y|} \end{array}$$ (24)

Where x and y represents the pixel rows and columns of the plaintext image (p(x,y)) respectively.

**Table 8:** Homogeneity analysis.

Plaintext images	Ref [66]	Ref [67]	Ref [68]	Ref [69]	Ref [70]	Ref [71]	Ref [72]	Ref [73]	Proposed
**Airport**	0.4936	0.4978	0.4606	0.5998	0.4679	0.4778	0.4978	0.4690	0.4532
**Tank**	0.4933	0.4977	0.4116	0.4999	0.4699	0.4898	0.4887	0.4766	0.4533
**Drone**	0.4899	0.4996	0.4663	0.4996	0.4697	0.4788	0.4763	0.4996	0.4500
**Military base**	0.4866	0.4896	0.4668	0.4986	0.4796	0.4861	0.4730	0.4986	0.4550
**Ground station**	0.4866	0.4866	0.4796	0.4701	0.4866	0.4700	0.4901	0.4896	0.4510

### 6.8 Contrast

Contrast analysis is used to identify the objects in an image. In an enciphered image, the randomness raises the contrast value. Higher contrast values imply better encryption. Mathematically, it can be calculated as [76]:

$$\begin{array}{c} Contrast=\sum_{q,r=0}{\left|q-r\right|}^{2}\gamma \left(q-r\right) \end{array}$$ (25)

Where *q* and *r* are the 8-bit gray level images and *γ*(*q* − *r*) is the gray level occurrence matrix. Contrast values for the proposed and the existing encryption algorithm are reported in Table 9. By analyzing such values, it can be seen that the proposed encryption algorithm works better than comparable schemes.

**Table 9:** Contrast analysis.

Plaintext images	Ref [66]	Ref [67]	Ref [68]	Ref [69]	Ref [70]	Ref [71]	Ref [72]	Ref [73]	Proposed
**Airport**	9.3986	9.6789	9.4689	9.9980	9.6487	9.7832	9.9613	9.9992	10.6989
**Tank**	9.8826	9.7910	9.8960	9.8741	9.9982	9.7966	9.9710	9.8761	10.2311
**Drone**	9.9784	9.7812	9.8850	9.8713	9.8775	9.8873	9.9970	9.9460	10.6790
**Military base**	9.9741	9.6780	9.7710	9.8820	9.8711	9.9462	9.9910	9.9784	10.3460
**Ground station**	9.8746	9.6692	9.9740	9.7601	9.7880	9.9970	9.9631	9.9631	10.3102

### 6.9 Energy

The term energy is used to find the amount of information present in the image [9]. More information present in an image shows that the image has more energy. Therefore, for strong encryption, it necessary that the encryption algorithm should be able to generate such type of ciphertext images in which minimum information can be visualized. As the plaintext images contain more information than the ciphertext images, the energy value for the plaintext image is always higher than that of the energy value of the ciphertext images [77]. Mathematically, energy can be represented as:

$$\begin{array}{c} Energy=\sum {\left(g,m\right)}^{2} \end{array}$$ (26)

Table 10 shows the comparison of different energy values corresponds to the plaintext images, existing scheme and the proposed encryption scheme. It can be analyzed from Table 10 that the proposed encryption algorithm can generate such ciphertext images which have fewer energy values than that of plaintext images and other existing schemes.

**Table 10:** Energy analysis.

Plaintext images	Ref [66]	Ref [67]	Ref [68]	Ref [69]	Ref [70]	Ref [71]	Ref [72]	Ref [73]	Proposed
**Airport**	0.0159	0.0158	0.0168	0.0169	0.0163	0.0162	0.0160	0.0160	0.0153
**Tank**	0.0162	0.0161	0.0159	0.0164	0.0161	0.0160	0.0163	0.0154	0.0151
**Drone**	0.0159	0.0159	0.0159	0.0160	0.0162	0.0161	0.0163	0.0164	0.0154
**Military base**	0.0159	0.0158	0.0158	0.0161	0.0163	0.0161	0.0167	0.0163	0.0153
**Ground station**	0.0161	0.0160	0.0164	0.0160	0.0162	0.0163	0.0165	0.0162	0.0154

### 6.10 Lossless analysis

To retrieve the exact pixel values of the plaintext image from the ciphertext image, the encryption algorithm must be lossless. To show the encryption algorithm is lossless or not, two different terms Peak signal to noise ratio (PSNR) and mean square error (MSE) are frequently used. Mathematically these terms can be represented as:

$$\begin{array}{c} MSE=\frac{1}{LM}\sum_{w=0}^{L-1}\sum_{t=0}^{M-1}{\left(O\left(w,t\right)-X\left(w,t\right)\right)}^{2} \end{array}$$ (27)

$$\begin{array}{c} PSNR=10\times log_{2}\frac{P_{max}^{2}}{MSE} \end{array}$$ (28)

PSNR and MSE are two opposite terms. PSNR is used to check the similarity index between the plaintext and ciphertext images. More the similarity between the plaintext and ciphertext image will result in the higher values of PSNR which is not required in image encryption. Therefore, an encryption algorithm having strong security always produced minimum PSNR values. In contrast, MSE is used to evaluate the difference between the two desired images. For strong encryption, the MSE value should be high. Maximum MSE value shows that the plaintext and ciphertext images are completely different from each other [78]. To evaluate the proposed encryption algorithm is lossless, PSNR values and MSE values are calculated which are shown in Tables 11 and 12 where it can be analyzed that the PSNR and MSE values for the proposed work are zero and infinity respectively. Whereas the existing schemes show the PSNR and MSE values other than zero and infinity which means that the comparable scheme cannot be used where exact pixel values are required to retrieve.

**Table 11:** MSE for loseless analysis.

Plaintext images	Ref [66]	Ref [67]	Ref [68]	Ref [69]	Ref [70]	Ref [71]	Ref [72]	Ref [73]	Proposed
**Airport**	5.68	8.91	6.86	5.13	3.18	9.98	3.67	6.64	0
**Tank**	6.97	9.93	5.68	3.90	4.54	9.78	4.31	3.67	0
**Drone**	3.67	8.54	4.16	3.48	3.66	3.61	2.61	4.10	0
**Military base**	7.97	6.87	9.18	8.99	3.97	3.99	3.37	4.36	0
**Ground station**	7.97	9.38	3.94	4.033	4.08	4.16	3.34	3.32	0

**Table 12:** PSNR for loseless analysis.

Plaintext images	Ref [66]	Ref [67]	Ref [68]	Ref [69]	Ref [70]	Ref [71]	Ref [72]	Ref [73]	Proposed
**Airport**	202.3581	209.9781	189.9831	198.3641	192.6798	216.9987	253.6713	196.378	∞
**Tank**	216.0146	218.3987	196.0166	207.1982	209.4930	207.1887	266.3014	186.3751	∞
**Drone**	207.8913	239.4680	207.6798	219.6782	207.1889	213.3368	228.3871	205.3781	∞
**Military base**	228.8712	216.0387	215.9780	205.7965	215.3687	207.7341	286.3746	206.3781	∞
**Ground station**	217.6715	216.3363	207.6985	229.9981	215.7319	227.1472	199.3741	199.6712	∞

### 6.11 Cropping attack analysis

**Fig 10:**
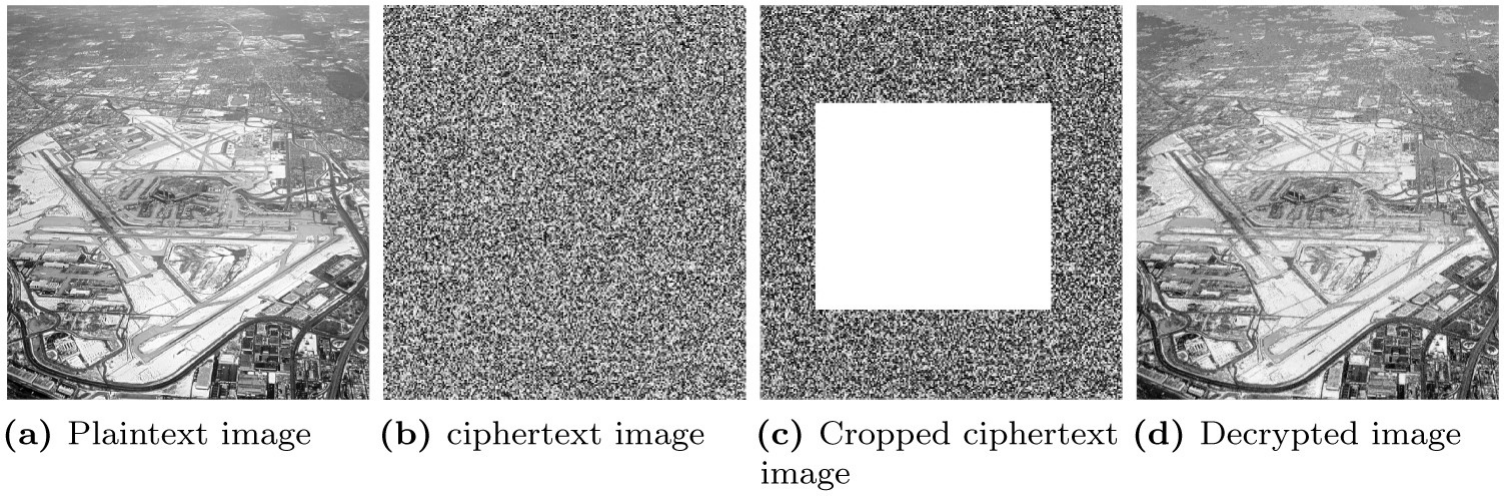
Cropping attack analysis. (a) Plaintext image, (b) ciphertext image, (c) Cropped ciphertext image, and (d) Decrypted image.

While sending the encrypted images to the ground station, it is possible that the attacker may crop the image to destroy the original information (which is encrypted in the ciphertext image). To gauge the performance of the proposed work in terms of cropping attack analysis, a portion of the ciphertext image is cropped and then send to the ground station. It is decrypted at the receiver end, and it is analyzed that if the attacker cropped the encrypted image, the proposed algorithm is still able to decrypt the original image with little loss of information. Fig 10 shows the cropping attack analysis in which Fig 10(a) shows the cropped version of the ciphertext image and Fig 10(b) shows the decrypted image which is retrieved from the cropped version of the encrypted image. It is clear from Fig 10 that the proposed encryption algorithm can resist the cropping attack analysis.

### 6.12 Noise attack analysis

There is another category of attack that attackers can launch to shatter the original information. To perform the noise attack analysis for the proposed encryption algorithm, salt (*S*) and pepper (*P*) noise is added in the ciphertext image using Eq (29):

$$\begin{array}{c} \text{Noisy}\;\text{ciphertext}\;\text{image}=S+P+C(i,j) \end{array}$$ (29)

**Fig 11:**
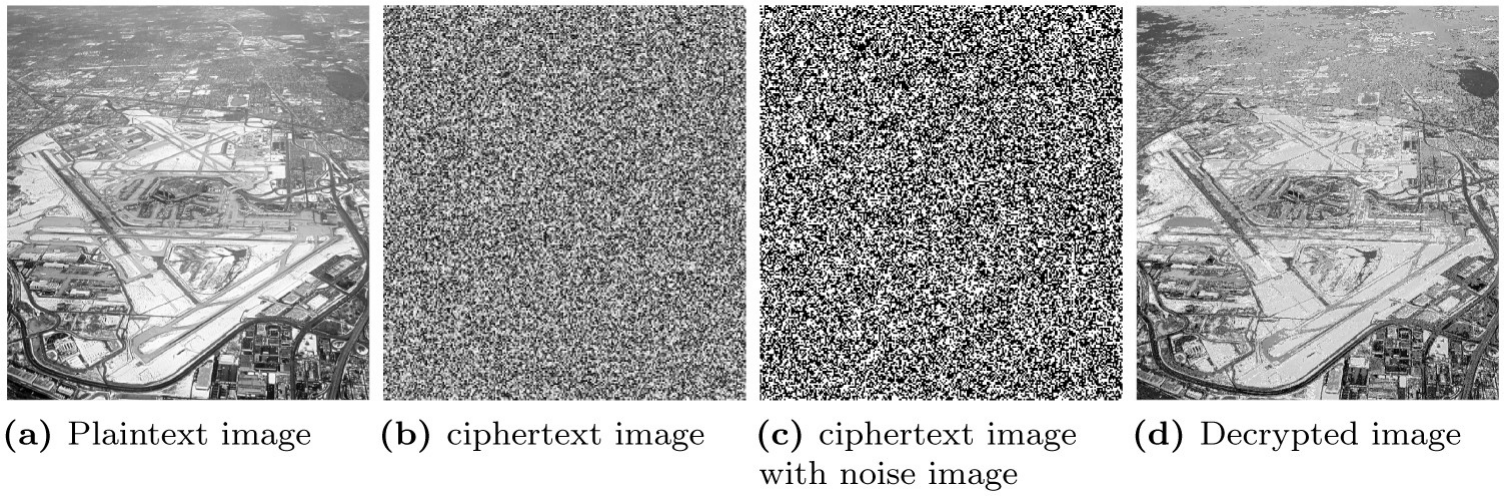
Noise attack analysis. (a) Plaintext image, (b) ciphertext image, (c) ciphertext image with noise image, and (d) Decrypted image.

Where *i*, *j* is the pixel position of the ciphertext image *C* in which the noise in the form of *S* and *P* is added. After the addition of noise in the ciphertext image, decryption is performed through the proposed decryption algorithm, and it is found that the information in the decrypted image can be clearly visualized as can be seen in Fig 11. Although there is a little noise in the decrypted image, but perceptually there is no difference between the original and ciphertext image.

### 6.13 Keyspace analysis

Keyspace analysis can be used to evaluate that the weather the encryption scheme can resists the brute force attack or not. Brute force attacks refer to the number of possible combinations of the security keys. For the strong and secure encryption algorithm, the key size should be large enough to resist the brute force attack [79]. According to Alvazari [80], the key size should be at least 10^100^. In the proposed work, there are four security keys (*x*_0_, *x*_1_, *r*_0_, *r*_1_) are used. As the sensitivity of the each key is 10^−15^, the key space of each key will be 10^+ 15^. This means the total key space for keys used in the proposed work will be 10^15*4^ which is approximately equal to 2^200^. Therefore, according to Alvazari’s criteria, our algorithm can resist the brute force attack.

### 6.14 Key sensitivity analysis

**Fig 12:**
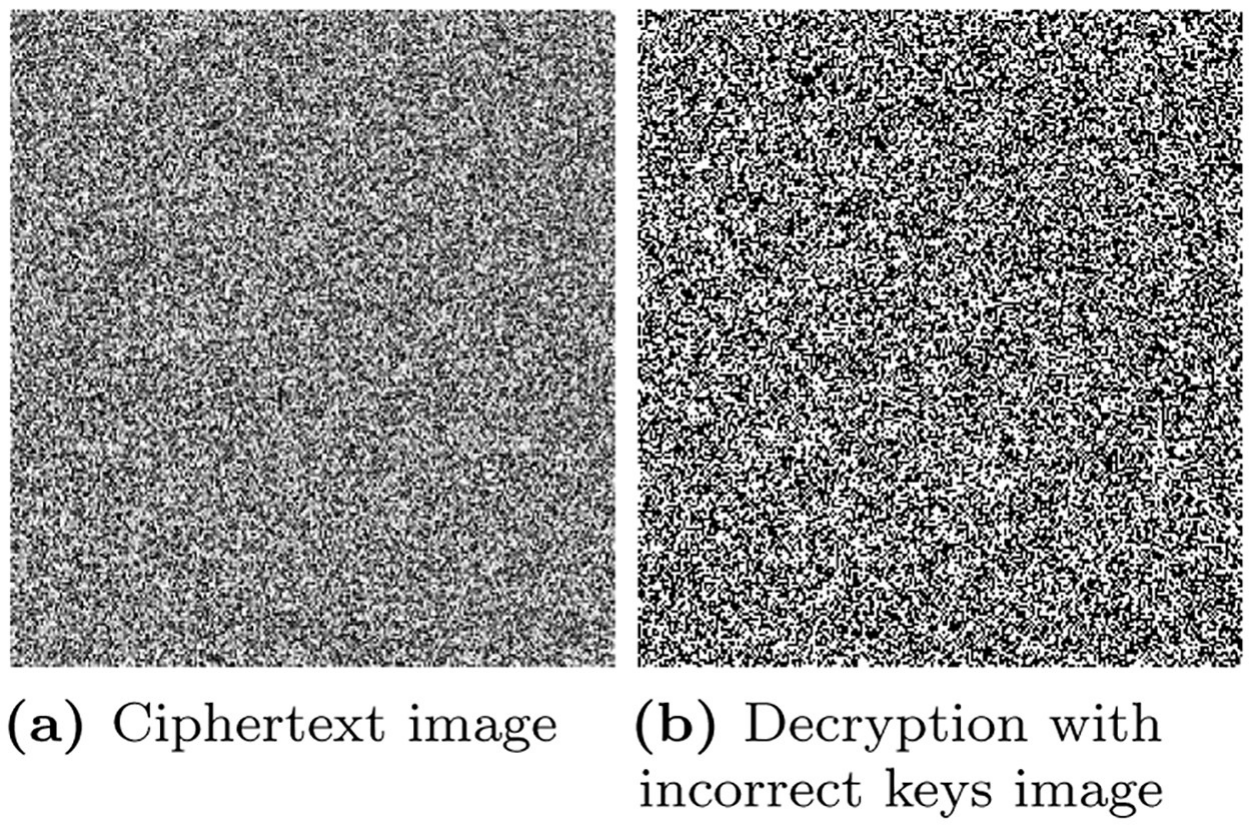
Key sensitivity analysis.

The security strength also depends on the keys which are used in the encryption algorithm. It refers to a small change in the security keys generate significant different ciphertext image. To prove the proposed encryption algorithm is a key sensitive, a tiny change (Δ = 10^−15^) made in the security keys (*r* and x_0_) I,e *r*′’ = *r* + delta, and $x^{{}_{0}^{\prime }}\prime =x_{0}+\text{delta}$. The modified keys ($X^{{}_{0}^{\prime }}$, *r*′) are then used to decrypt the plaintext image. The resultant decrypted image is shown in Fig 12(b). It can be seen that a tiny change in the security can led to decryption failure.

### 6.15 MSE and PSNR analysis

MSE is used to calculate the error between the plaintext and ciphertext image. For strong encryption, MSE values should be as high as possible. While PSNR is the reciprocal of MSE. Therefore, if the encryption algorithm offers strong security, the PSNR between the plaintext and ciphertext image will be low. These two metrics can be calculated using Eqs 27 and 28 respectively. In Table 13, MSE and PSNR values are displayed for the proposed and existing encryption schemes. The displayed values reveal that the proposed scheme works better than the existing encryption schemes in terms of PSNR and MSE.

**Table 13:** MSE and PSNR analysis.

Plain images	Proposed algorithm		Ref [68]		Ref [69]		Ref [70]	
	PSNR	MSE	PSNR	MSE	PSNR	MSE	PSNR	MSE
**Airport**	10.9871	6579	15.3710	2679	17.9790	3511	21.9786	2973
**Tank**	10.9731	5988	17.5899	3033	20.6681	36536	20.3681	3066
**Drone**	10.6687	5866	14.6582	3136	21.6797	3496	19.6681	3079
**Military base**	11.6792	6431	13.6497	3386	20.9987	3596	20.3676	3367
**Ground station**	10.3798	6226	12.6797	3267	19.9990	3193	19.9965	3567

### 6.16 Sensitivity analysis

Attackers often make a small change in plaintext image and encrypt it before and after this change. By comparing both the encrypted images, they find the relationship between such encrypted images. This attack is known as differential attack. To resists this attack, two well-known metrics number of pixels’ change rate (NPCR) and unified average changing intensity can be used. NPCR and UACI can be calculated using Eqs 30 and 31 respectively [81].

$$\begin{array}{c} NPCR=\frac{\sum_{q,r}D\left(q,r\right)}{A\times B}\times 100\% \end{array}$$ (30)

$$\begin{array}{c} UACI=\frac{1}{A\times B}\sum_{q,r}\frac{|C_{1}\left(q,r\right)-C_{2}\left(q,r\right)|}{255}\times 100\% \end{array}$$ (31)

Where, *A* and *B* represents the pixel rows and columns of the image. *C*_1_ and *C*_2_ are the two ciphertext images whose corresponding plaintext images have only pixel change. The difference matrix *D*(*q*, *r*) can be determined by *C*_1_ and *C*_2_. If *C*_1_ = *C*_2_, then *D*(*q*, *r*) = 0; otherwise. The larger the value of UACI and NPCR, the better encryption. Apart from UACI and NPCR, another metric known as Mean Absolute Error (MAE) is used to evaluate the performance of the encryption against differential attacks. Mathematically, it can be represented as;

$$\begin{array}{c} MAE=\frac{1}{A\times B}\sum_{q=1}^{A}\sum_{r=1}^{B}\left|C_{1}\left(q,r\right)-P\left(q,r\right)\right| \end{array}$$ (32)

Where, *C*(*q*, *r*) and *P*(*q*, *r*) are the ciphertext and plaintext image, respectively. For better encryption, the value of MAE must be larger. The UACI, NPCR and MAE for the proposed and existing encryption schemes values are displayed in Tables 14–16 respectively. It can be analyzed from these values that the proposed encryption scheme works better and can resist the differential attack.

**Table 14:** UACI analysis.

Plaintext images	Ref [66]	Ref [67]	Ref [68]	Ref [69]	Ref [70]	Ref [71]	Ref [72]	Ref [73]	Proposed
**Airport**	33.4050	33.5972	33.4125	33.2831	33.1831	33.5831	33.5831	33.3831	33.6554
**Tank**	33.5050	33.4972	33.4125	33.5831	33.5831	33.5831	33.5831	33.3831	33.6654
**Drone**	33.4050	33.4972	33.5125	33.3831	33.3831	33.5831	33.3831	33.3831	33.6754
**Airport**	33.4050	33.4972	33.5125	33.5831	33.3831	33.3831	33.5831	33.3831	33.6532
**Military base**	33.4050	33.2972	33.2125	33.5831	33.5831	33.5831	33.3831	33.2831	33.6111
**Airport**	33.4050	33.4972	33.3125	33.5831	33.3831	33.3831	33.3831	33.1831	33.6330
**Ground station**	33.4050	33.4972	33.5125	33.5831	33.3831	33.3831	33.3831	33.2831	33.6390

**Table 15:** NPCR analysis.

Plaintext images	Ref [66]	Ref [67]	Ref [68]	Ref [69]	Ref [70]	Ref [71]	Ref [72]	Ref [73]	Proposed
**Airport**	33.4050	33.4972	33.2125	33.3831	33.3831	33.3831	33.3831	33.3831	33.6532
**Tank**	33.4050	33.2972	33.4125	33.5831	33.5831	33.5831	33.5831	33.5831	33.6615
**Drone**	33.5050	33.2972	33.4125	33.3831	33.3831	33.5831	33.3831	33.5831	33.6723
**Airport**	33.5050	33.4972	33.5125	33.5831	33.5831	33.3831	33.3831	33.2831	33.6899
**Military base**	33.4050	33.4972	33.4125	33.3831	33.3831	33.3831	33.5831	33.5831	33.6783
**Airport**	33.5050	33.4972	33.4125	33.3831	33.3831	33.3831	33.3831	33.3831	33.6063
**Ground station**	33.4050	33.4972	33.4125	33.3831	33.3831	33.3831	33.3831	33.2831	33.6166

**Table 16:** MAE analysis.

Plaintext images	Ref [66]	Ref [67]	Ref [68]	Ref [69]	Ref [70]	Ref [71]	Ref [72]	Ref [73]	Proposed
**Airport**	2687	2366	2454	2963	2999	2876	2331	2677	3668
**Tank**	2879	2364	2100	2336	2794	2650	2116	2998	3570
**Drone**	2973	2347	2017	2367	2496	2887	2778	2736	3321
**Airport**	2964	2376	2886	2640	2641	2007	2666	2987	3336
**Military base**	2679	2446	2665	2314	2007	2667	2476	2766	3166
**Airport**	2793	2467	2366	25559	2367	2674	2554	2778	3369
**Ground station**	2674	2116	2779	2466	2311	2555	2491	2676	3560

### 6.17 NIST-800-22

To assess the performance of random sequences, NIST-800-22 was designed. Based on the findings of the NIST test, one can identify whether the chaotic sequence is appropriate for use in a cryptographic method or not. In addition to the frequency and run tests, the NIST-800-22 contains thirteen more test methods, which include random excursions, approximate entropy tests, etc. The *p* value may be used to determine the randomness of a test sequence. The sequence is random if *p* ≥ 0.01, otherwise *p* < 0.01 shows that the sequence is not random. Moreover, the randomness of the sequence improves when the *p* value is larger. According to Table 17, the generated sequences in the proposed encryption algorithm pass all random tests.

**Table 17:** NIST analysis.

Test methods	P-value	Result
Longest Run	0.5988	Cleared
Random Excursions	0.5130	Cleared
Nonoverlapping Template	0.5316	Cleared
Overlapping Template	0.4961	Cleared
Linear Complexity	0.4689	Cleared
Random Excursions Varient	0.5013	Cleared
FFt	0.5316	Cleared
Ranks	0.4770	Cleared
Frequency	0.5697	Cleared
Runs	0.5336	Cleared
Cumulative Sum	0.4986	Cleared
Serial Test	0.5110	Cleared
Block Frequency	0.5987	Cleared
Maurer’s Universal	0.6761	Cleared
Approximate Entropy	0.4798	Cleared

### 6.18 Encryption computational time analysis

Apart from security analysis, time analysis is a critical metric for evaluating the performance of an encryption algorithm. The proposed encryption scheme is specifically designed for UAV applications where the encryption scheme must be time-efficient. For the encryption computational analysis, platform having a specification and 8GB of RAM is considered. Moreover, a built-in command in MATLAB called tic toc is used to calculate the processing time of the encryption and decryption. The platform to calculate the computational complexity for the proposed and existing work is kept the same. The processing time of the proposed and existing schemes is given in Table 18, and it can be seen that the proposed scheme is more suitable than the existing schemes for real-time applications.

**Table 18:** Computational time analysis (sec).

Plaintext images	Ref [66]	Ref [67]	Ref [68]	Ref [69]	Ref [70]	Ref [71]	Ref [72]	Ref [73]	Proposed
**Airport**	2.5813	0.569	0.3381	0.6649	0.8644	0.3973	0.6690	2.9793	0.0058
**Tank**	2.6610	0.5812	0.2871	0.8671	0.6112	0.6971	0.8668	2.6352	0.0018
**Drone**	3.7710	0.6187	0.8371	0.7741	0.6478	0.4972	0.6541	3.8733	0.0041
**Military base**	2.6792	0.9912	0.2987	0.6178	0.9349	0.8963	0.4896	2.6970	0.0043
**Ground station**	2.6913	0.7613	0.6175	0.8360	0.6336	0.7339	0.9799	3.6934	0.0041

### 6.19 Ciphertext only attack

In this case, the attacker has access to only the ciphertext image and if he does not know which encryption algorithm is used to generate that ciphertext image, this attack is the most difficult to successfully execute to decrypt the plaintext image, as it is also stated in [82]. In our case, the ciphertext image is generated using the confusion-diffusion mechanism, which is achieved by employing DWT, bit-plane extraction, XOR operation, and a few random sequences. According to the statistical analysis such as entropy, contrast, energy, and correlation, it might be very difficult to recover the plaintext image from the only ciphertext image. Therefore, the proposed encryption scheme can resist this attack.

### 6.20 Known plaintext attack

In this case, the attackers have few plaintext images and their ciphertext images. Based on the knowledge of plaintext-ciphertext pairs, the attackers try to find the secret keys used in the encryption algorithm so that a correct version of the original message can be recovered. To show the robustness of the proposed encryption algorithm against this attack, key-space analysis plays a vital role. From the key-space analysis, it can be seen that it is nearly impossible to find the correct encryption keys in a meaningful time slot. Therefore, the proposed encryption algorithm is resistive to known plaintext attack.

### 6.21 Chosen plaintext attack

To execute this attack successfully by the attack or cryptanalyst, several plaintext images are encrypted with the encryption algorithm to find the secret keys. From the key-sensitivity analysis, one can see that the secret keys used in the proposed encryption algorithm are sensitive. Even a minor change can make a huge difference in the decrypted image. Therefore, according to the key sensitive analysis, the proposed scheme can resist this attack.

### 6.22 Chosen ciphertext attack

In this case, the cryptanalyst has different ciphertext images and tries to decrypt them with the decryption algorithm. Again, the purpose of this attack is to find the original keys that are used to encrypt the meaningful message. The random sequences used in the proposed work are based on the initial conditions of the chaotic maps, which are highly sensitive, as shown in the key-sensitivity analysis. Therefore, according to such analysis, the proposed work can withstand a chosen ciphertext attack.

## 7 Conclusion

The proposed encryption scheme is especially designed for usage in real-time applications. When it comes to real-time applications, they always need less time for encryption and computing. The bit-plane extraction technique and DWT are included in the proposed scheme in order to make it appropriate for use in real-time applications. Only the most significant bit bit planes and low-frequency sub-bands are taken into consideration throughout the encryption process. This is because the majority of the plaintext information is located in these bit planes and sub-bands. In addition to this, several chaotic maps are incorporated in order to increase its level of security. These chaotic maps produce unpredictable sequences and random images for the purposes of confusion and diffusion. In order to evaluate how well the proposed scheme works, a number of security analysis, including entropy, energy, correlation, PSRN, and MSE are carried out. The results of such analysis are compared with those of the existing scheme, which demonstrates that the proposed scheme works more effectively than the existing schemes. In addition to the statistical analysis, several security attacks, including cropping, noise, and brute force attacks, are carried out on encrypted images that are encrypted using the proposed encryption scheme. It is demonstrated that the proposed encryption algorithm is capable of withstanding such attacks.
